# Effect of Voice Attractiveness and Group Identity in an Ultimatum Game

**DOI:** 10.1002/pchj.70013

**Published:** 2025-03-25

**Authors:** Junchen Shang, Kaiyin Zhong, Rui Shi

**Affiliations:** ^1^ Department of Medical Humanities, School of Humanities Southeast University Nanjing China; ^2^ School of Economics and Management Yanshan University Qinhuangdao China

**Keywords:** beauty premium, fairness, group identity, ultimatum game, voice attractiveness

## Abstract

This study examined how voice attractiveness and group identity influence ultimatum decisions. Attractive voices only increased acceptance of 8:2 offers, suggesting a weak beauty premium effect. In‐group proposers' unfair offers also had elevated acceptance, supporting Social Identity Theory.

## 1 Introduction

Prior research has demonstrated the existence of the beauty premium effect in voices. Individuals accepted more unfair offers from attractive‐voiced proposers in an ultimatum game (Shang and Liu 2023). Furthermore, voice attractiveness ratings are moderated by group membership, because men evaluated in‐group fertile women's voices as more attractive than nonfertile ones. However, this preference does not extend to out‐group (Tidwell et al. 2017), suggesting voice attractiveness and group identity are important in human evolution. The role of group identity on decisions is widely examined. Social Identity Theory elucidates that in‐group members get more positive evaluations and fewer punishments for unfair actions than out‐group members (McAuliffe and Dunham 2016). However, Norm‐Focused Theory posits the black sheep effect that norms violations by in‐group members induce stronger disgust and harsher punishment than out‐group members. No research tested how voice attractiveness and group identity simultaneously impact decision‐making, especially for decisions toward unfairness. As Inequity Aversion Theory suggested, people not only care about their own interests but also attach importance to fair distribution (Fehr and Schmidt 1999). Therefore, this study investigated how voice attractiveness, group identity, and fairness worked in concert in ultimatum decisions. It would not only provide more explanations for complex social behaviors, but also provide guidance to promote fairness in real life.

To our knowledge, no existing research explored the influence of the three factors together in decision‐making, and we make a hypothesis based on Tidwell et al. (2017) that there may be an interaction between voice attractiveness and group identity, especially in unfair offers. As an exploratory study, we do not have specific hypotheses about the simple effects of each variable.

## 2 Methods

Following ethical approval by the Ethics Committee of the Psychology Research Center at Southeast University, 55 female students (*M*_age_ = 23.85 years, SD = 3.05) from Southeast University were enrolled in the experiment. Written informed consent was obtained from all participants before their involvement.

The present study employed a 2 (voice attractiveness: attractive, unattractive) × 5 (fairness: 9:1, 8:2, 7:3, 6:4, 5:5) × 2 (group identity: in‐group, out‐group) within‐participant design. Twelve attractive and twelve unattractive male voices of 2040 ms were used (Shang and Liu 2022). Each voice pronounces three nonsense syllables (/i/, /a/, /ou/). These voices were divided into four groups based on attractiveness ratings, with six voices in each group. To ensure attractiveness ratings did not differ between in‐group and out‐group conditions, a two‐way ANOVA (voice attractiveness × group identity) was conducted on attractiveness ratings. Only the main effect of voice attractiveness was significant, *p* < 0.001. Detailed statistics including the acoustic parameters are in Supporting Information Data (S1).

This study implemented a two‐phase procedure beginning with group classification. Based on the Minimal Group Paradigm, which is a sham personality test described by Wu et al. (2015), participants were arbitrarily assigned to red/blue personality groups. In the subsequent interaction phase, participants engaged in bargaining tasks where the proposer determined how to divide ¥10 between himself and the participant, with the latter retaining decision authority to accept/reject the proposed allocation. For the improvement of group manipulation, participants were informed the higher total‐income group would receive supplementary monetary rewards (Rilling et al. 2008). The experimental sequence initiated with a central fixation displayed for 400–600 ms, followed by a proposer' voice with red/blue background (2040 ms). After a 200–300 ms interval, a proposal to split ¥10 appeared until the participant responded. Finally, a 2000‐ms outcome was shown after a 200–300 ms interval. Each voice was matched once with each allocation, with 120 trials presented in a pseudo‐random sequence that the same voice was not shown in successive trials. Before the experiment commenced, there were 20 practice trials. After the experiment, participants evaluated the voice attractiveness using a 7‐point scale (1 = *unattractive*, 7 = *attractive*).

## 3 Results

**FIGURE 1:**
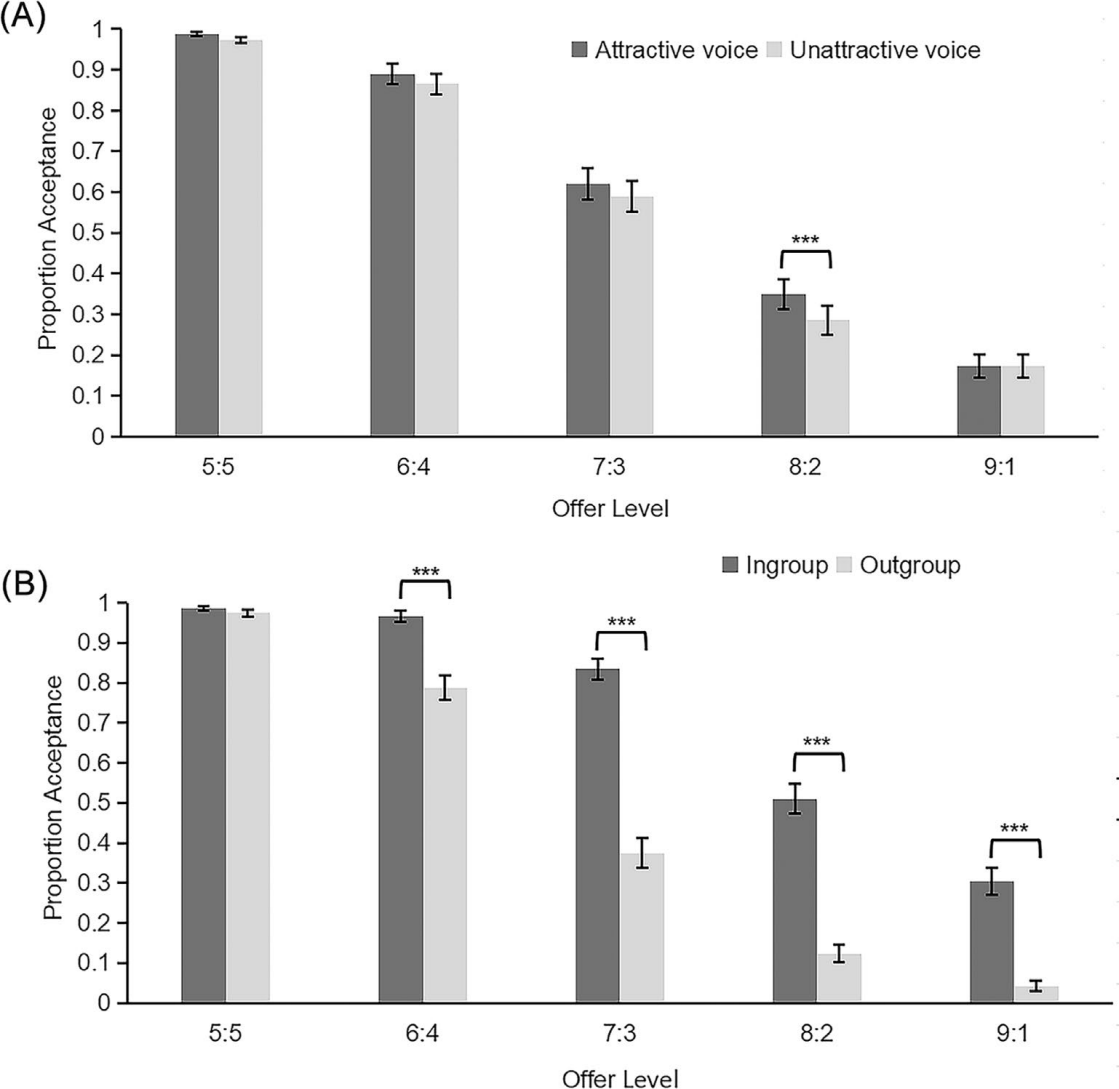
(A) Mean proportion acceptance as a function of offer fairness and voice attractiveness. (B) Mean proportion acceptance as a function of offer fairness and group identity. Error bars represent standard errors. ***p < 0.001.

Three‐way repeated measures ANOVA was performed on acceptance rate. Greenhouse–Geisser correction was applied for sphericity departures. All post hoc analyses were Bonferroni‐corrected. The main effects of voice attractiveness, fairness, and group identity were significant, *F*s ≥ 8.87, *p*s ≤ 0.004. The interaction between voice attractiveness and fairness was significant (Figure 1A), *F*(3, 165) = 2.866, *p* = 0.037, *η*_*p*_^2^ = 0.05. Simple effect analysis showed acceptance rate of unfair allocation (8:2) from attractive proposers was significantly higher than that from unattractive proposers, *F*(1, 54) = 15.202, *p* < 0.001, *η*_*p*_^2^ = 0.22. Nevertheless, this attractiveness effect was absent at other allocations, *F*s ≤ 3.846, *p*s ≥ 0.055. The interaction between group identity and fairness was significant (Figure 1B), *F*(3, 170) = 25.161, *p* < 0.001, *η*_*p*_^2^ = 0.318. Simple effect analysis showed acceptance rate of all unfair allocations from in‐group proposers was significantly higher than that from out‐group proposers, *F*s ≥ 19.846, *p*s < 0.001. Other effects were not significant, *F*s ≤ 1.738, *p*s ≥ 0.193.

Post‐experimental ratings indicated attractive voices (*M* = 4.72, SD = 0.75) were rated as more attractive than unattractive voices (*M* = 2.86, SD = 0.78), *t* (22) = 5.947, *p* < 0.001, 95% CI = [1.21, 2.52].

## 4 Discussion

Beauty premium occurred only in hard decisions, where participants were more inclined to accept a more moderately unfair offer 8:2 from attractive proposers, whereas this effect was absent at other offers. This is partly consistent with prior research (Shang and Liu 2023). Nevertheless, participants were more objective with a moderately unfair offer 7:3 and an extremely unfair offer 9:1. The acceptance rate for offer 9:1 (17.3%) indicated a potential floor effect. The reason why offer 7:3 showed no attractiveness effect could be participants' strategy to earn additional group reward. Voice attractiveness's weak effect may stem from a small rating difference (1.87) between attractive and unattractive voices. Another explanation is that visual cues of group identity provide more salient information than voice cues for decisions. Participants are more easily to make decisions under in‐group condition and are not likely to consider voices. Although the three‐way interaction was not significant, analyses under controlled visual cue conditions showed a stronger effect of voice attractiveness under out‐group condition (see Data S1).

Participants showed greater acceptance of all unfair allocations from in‐group proposers. This supported Social Identity Theory (McAuliffe and Dunham 2016), indicating the in‐group favoritism. We did not detect black sheep effect because of the competitive decision context. Participants considered additional group reward by accumulating income; therefore, they rejected more unfair allocations from out‐group.

## Ethics Statement

The research was approved by the Ethics Committee of the Psychology Research Center at Southeast University. Each participant provided written informed consent.

## Conflicts of Interest

The authors declare no conflicts of interest.

## Supporting information


Data S1.
