# Supplementary material for: Effect of Voice Attractiveness and Group Identity in an Ultimatum Game
Source: Psych J. 2025 Mar 25;14(3):460–2. doi: 10.1002/pchj.70013 (PMC12133227; doi:10.1002/pchj.70013)
Supplement: Supplementary file 1 — Data S1. [file PCHJ-14-460-s001.docx]

**Part 1 Voice selection and acoustic parameter analyses**

*The voice stimuli were selected from Ferdenzi et al. (2015)’s database. There were 111 voice samples (61 female voices, 50 male voices, M_age_ = 22.9 years, SD = 4.3), with each voice sample reading three neutral vowels: /i/, /a/, /ou/. The duration of all voice samples was adjusted to 2040 ms, and the sound intensity was standardized to 70 dB. Shang & Liu (2022) recruited 58 college students (18 males, M_age_ = 21.60 years, SD = 2.43) to rate the attractiveness of the voices on a 7-point scale (1 = very unattractive, 7 = very attractive). After sorting the voice stimuli based on the mean attractiveness ratings, and considering both the quantity of voice stimuli and the needs of this study, we ultimately selected 12 attractive and 12 unattractive male voices (see* ***Table 1*** *for the descriptive statistics of the ratings). From the remaining materials, we randomly selected 2 attractive and 2 unattractive male voices for the practice phase.*

Table 1. Means and standard deviations of ratings of voice attractiveness in different groups (M ± SD).

|  | **In-group** | | **Out-group** | |
| --- | --- | --- | --- | --- |
|  | ***M*** | ***SD*** | ***M*** | ***SD*** |
| Attractive voices | 5.04 | 0.41 | 5.00 | 0.30 |
| Unattractive voices | 2.74 | 0.63 | 2.80 | 0.45 |

*According to the experimental design, we randomly assigned the 24 voices into four groups: attractive voices-in-group, unattractive voices-in-group, attractive voices-out-group and unattractive voices-out-group. Each group contains 6 voices. A two-way ANOVA was conducted on the attractiveness ratings of the 24 voices, and the results showed that: The main effect of voice attractiveness was significant, F(1,56) = 141.49, p < .001, η_p_² = .876. The main effect of group identity was not significant, F(1,56) = 0.005, p = .946. The interaction between voice attractiveness and group identity was not significant, F(1,56) = 0.063, p = .805.*

*Moreover, the acoustic parameters of attractive voices and unattractive voices were calculated using Praat software (Boersma & Weenink, www.praat.org) and were compared using paired t-tests (as shown in* ***Table 2****). Previous studies suggested that there is a correlation between F0 and voice attractiveness (Feinberg et al., 2006). Adult women prefer male voices with lower-pitched and lower-F0 (Feinberg et al., 2005). The results were consistent with previous research that the F0 of male voices with attractiveness is higher than that of male voices with unattractiveness (Pisanski & Rendall, 2011). Additionally, the f3 value of attractive male voices is significantly higher than that of unattractive male voices. Conversely, the HNR value of unattractive male voices is significantly higher than that of attractive male voices (Shang & Liu, 2022).*

Table 2. Means(and standard deviations) and acoustic differences between attractive and unattractive male voices.

|  | **Male voices** | | | | |
| --- | --- | --- | --- | --- | --- |
|  | **Attractive** | **Unattractive** | ***t*** | ***p*** | **Cohen’s *d*** |
| F0 | 126.12(17.13) | 142.73(29.96) | -1.67 | 0.113 | -0.68 |
| f1 | 616.82(79.50) | 611.62(92.93) | 0.15 | 0.884 | 0.06 |
| f2 | 1613.80(54.06) | 1605.12(64.33) | 0.36 | 0.724 | 0.15 |
| f3 | 2796.81(67.69) | 2710.47(99.68) | **2.48** | **0.021** | 1.01 |
| f4 | 3701.71(81.51) | 3646.27(99.85) | 1.49 | 0.151 | 0.61 |
| Df | 1028.29(37.76) | 1011.55(48.42) | 0.95 | 0.355 | 0.39 |
| Pf | 0.22(0.49) | -0.22(0.77) | 1.66 | 0.111 | 0.68 |
| Jitter | 0.02(0.01) | 0.02(0.01) | 0.55 | 0.589 | 0.01 |
| Shimmer | 0.11(0.04) | 0.10(0.02) | 1.01 | 0.324 | 0.32 |
| HNR | 10.41(1.52) | 11.83(1.58) | **-2.25** | **0.035** | -0.92 |

*F0, fundamental frequency in Hz; f1–f4, formant frequencies in Hz; Df, formant dispersion in Hz; Pf, formant position; Jitter, variation of pitch in µs; Shimmer, variation of energy in dB; HNR, harmonic-to-noise ratio in dB.*

*References:*

*Ferdenzi, C., Delplanque, S., Mehu-Blantar, I., Da Paz Cabral, K. M., Domingos Felicio, M., & Sander, D. (2015). The Geneva faces and voices (GEFAV) database. Behavior research methods, 47, 1110–1121.*

*Feinberg, D. R., Jones, B. C., Little, A. C., Burt, D. M., & Perrett, D. I. (2005). Manipulations of fundamental and formant frequencies influence the attractiveness of human male voices. Animal behaviour, 69(3), 561–568.*

*Feinberg, D. R., Jones, B. C., Smith, M. L., Moore, F. R., DeBruine, L. M., Cornwell, R. E., ... & Perrett, D. I. (2006). Menstrual cycle, trait estrogen level, and masculinity preferences in the human voice. Hormones and behavior, 49(2), 215–222.*

*Pisanski, K., & Rendall, D. (2011). The prioritization of voice fundamental frequency or formants in listeners’ assessments of speaker size, masculinity, and attractiveness. The Journal of the Acoustical Society of America, 129(4), 2201–2212.*

*Shang, J., & Liu, Z. (2022). The “beauty premium” effect of voice attractiveness of long speech sounds in outcome-evaluation event-related potentials in a trust game. Frontiers in psychology, 13, 1010457.*

**Part 2 Analyses separately based on in-group and out-group**

*We further analyze the experimental data under controlled visual cue conditions by dividing the experimental data into two parts: the in-group and the out-group.*

*2 (voice attractiveness: attractive; unattractive) × 5 (fairness: 5:5, 6:4, 7:3, 8:2, 9:1) repeated measures analysis of variance (ANOVA) was performed on the acceptance rate under the in-group condition. Greenhouse–Geisser correction was used when the assumption of sphericity was not met. The main effect of voice attractiveness was not significant, F(1, 54) = 1.946, p = .169, η_p_^2^ = .035; The main effect of fairness was significant, F(2, 120) = 100.317, p < .001, η_p_^2^ = .65. Except for the insignificant difference between the 5:5 and 6:4 offers, all other pairwise combinations of offers showed significant differences, ps < .001. The interaction between voice attractiveness and fairness was significant, F(3, 166) = 2.918, p = .034, η_p_^2^ = .051. Simple effect analysis showed acceptance rate of unfair allocation (8:2) from attractive proposers (M = 0.545, SD = 0.052) was significantly higher than that from unattractive proposers (M = 0.476, SD = 0.056), F(1, 54) = 9.051, p < .05, η_p_^2^= .144.*

*2 (voice attractiveness: attractive; unattractive) × 5 (fairness: 5:5, 6:4, 7:3, 8:2, 9:1) repeated measures analysis of variance (ANOVA) was performed on the acceptance rate under the out-group condition. Greenhouse–Geisser correction was used when the assumption of sphericity was not met. The main effect of voice attractiveness was significant, F(1, 54) = 9.432, p < .05, η_p_^2^ = .149. The acceptance rate of offers from attractive proposers (M = 0.479, SD = 0.023) was significantly higher than that from unattractive proposers (M = 0.442, SD = 0.019). The main effect of fairness was significant, F(3, 139) = 188.191, p < .001, η_p_^2^ = .777. All pairwise combinations of offers showed significant differences, ps < .001. The interaction between voice attractiveness and fairness was not significant, F(3, 160) = 1.251, p = .293, η_p_^2^ = .023.*
